# Altered mean apparent propagator-based microstructure and the corresponding functional connectivity of the parahippocampus and thalamus in Crohn’s disease

**DOI:** 10.3389/fnins.2022.985190

**Published:** 2022-09-20

**Authors:** Yage Qiu, Qingshang Li, Dongmei Wu, Yiming Zhang, Jiahui Cheng, Zhijun Cao, Yan Zhou

**Affiliations:** ^1^Department of Radiology, Renji Hospital Affiliated to Shanghai Jiao Tong University School of Medicine, Shanghai, China; ^2^Department of Gastroenterology, Renji Hospital Affiliated to Shanghai Jiao Tong University School of Medicine, Shanghai, China; ^3^Department of Gastroenterology, Huadong Hospital, Shanghai Medical College, Fudan University, Shanghai, China; ^4^Shanghai Key Laboratory of Magnetic Resonance, East China Normal University School of Physics and Electronics Science, Shanghai, China

**Keywords:** mean apparent propagator, diffusion-weighted imaging, resting-state magnetic resonance imaging, functional connectivity, Crohn’s disease

## Abstract

Crohn’s disease (CD) is a chronic and relapsing inflammatory bowel disorder that has been shown to generate neurological impairments, which has the potential to signify disease activity in an underlying neurological manner. The objective of this study was to investigate the abnormalities of brain microstructure and the corresponding functional connectivity (FC) in patients with CD, as well as their associations with disease condition. Twenty-two patients with CD and 22 age-, gender-, and education-matched healthy controls (HCs) were enrolled in this study. All subjects underwent mean apparent propagator (MAP)-MRI and resting-state functional magnetic resonance imaging (MRI) (rs-fMRI) data collection. Each patient was evaluated clinically for the condition and duration of the disease. The MAP metrics were extracted and compared between two groups. Pearson’s correlation analysis was conducted to determine the relationship between disease characteristics and significantly abnormal MAP metrics in the CD group. Regions of interest (ROIs) for ROI-wise FC analysis were selected based on their correlation with MAP metrics. Results showed that multiple brain regions, including the parahippocampus and thalamus, exhibited statistically significant differences in MAP metrics between CD patients and HCs. Additionally, CD patients exhibited decreased FC between the left parahippocampus and bilateral thalamus, as well as the right parahippocampus and bilateral thalamus. The findings of this work provide preliminary evidence that structural abnormalities in the parahippocampal gyrus (PHG) and thalamus, as well as decreased FC between them, may reflect the degree of inflammatory of the disease and serve as brain biomarkers for evaluating CD activity.

## Introduction

Crohn’s disease (CD) is an inflammatory bowel disease (IBD) that is chronic and relapsing and may affect any portion of the digestive tract. In recent years, the disease incidence of CD in China has surged in tandem with the urbanization of Asian countries, population mobility, and dietary structure changes (Ng et al., 2017; Torres et al., 2017). The disease condition typically requires lifetime medication and supportive care on account of relapsing digestive tract inflammation and recurring intestinal or extraintestinal symptoms, greatly contributing to the poor life quality of CD patients (Torres et al., 2017). It has been proved that there are white matter (WM) lesions and neurological impairments existing in CD patients’ brains, which might represent an uncommon extraintestinal manifestation of the disease (Geissler et al., 1995). Given that no apparent brain structure abnormalities were found by conventional magnetic resonance imaging (MRI), more sophisticated imaging techniques and methodologies are required to study brain abnormalities underlying extraintestinal symptoms and to aid clinical diagnosis based on identified biomarkers (Geissler et al., 1995; Bonaz and Bernstein, 2013; Gracie et al., 2018).

Although the classical tensor model in diffusion tensor imaging (DTI) appears to be a sensitive tool for studying WM changes, it comes with a significant modeling constraint. That is, this method is predicated on the presumption that water diffusion in the brain is unrestricted and follows Gaussian distribution (Lee et al., 2018). However, as a matter of fact, water molecule diffusion often follows a non-Gaussian distribution in complex biological tissues owing to the presence of cell membranes, organelles, and liquid compartments (Khairnar et al., 2017). These microstructures cannot be adequately described by the tensor model, and biases may be introduced in the corresponding diffusion metrics in the meantime. Mean apparent propagator (MAP)-MRI, a newly developed diffusion model, has the potential to circumvent this limitation. The MAP-MRI model makes no prior presuppositions regarding the behavior of water diffusion in tissues (Hosseinbor et al., 2013; Olson et al., 2019). Instead, it is based on q-space sampling (Özarslan et al., 2013) and measures the probability density function of spin displacements in complex microstructures of brain tissue to examine the dispersion distribution of water molecules (Avram et al., 2016). This method offers a more comprehensive, delicate, and accurate tissue characterization than the tensor model by quantifying the non-Gaussian nature of the diffusion process, reflecting physiologically significant microstructural characteristics with a higher degree of neuroanatomical specificity (Özarslan et al., 2013; Olson et al., 2019).

It is salient to acknowledge that the vast majority of previous research has focused on either brain structure or brain function. To our knowledge, very few studies provide information on both brain structure and function in the CD sample (Kornelsen et al., 2020; Thomann et al., 2021), mostly using separate analysis approaches. While beyond doubt useful, such approaches may overlook vital sources of intermodal information. Yet, such information is essential for directly detecting potential links and similarities between patterns of brain structure and function in a specific population. Using concurrent multimodal MRI techniques, such as advanced diffusion model establishment and resting-state functional MRI (rs-fMRI) analysis, it is possible to detect co-altered patterns of brain changes that may be partially missed by conventional separate analyses.

In the meantime, few researches have examined the relationship between brain structure/function regulation and the inflammatory activity level of the disease, mostly focusing instead on association with cognitive function, mood, or pain scale. The dysfunction of the brain-gut axis is believed to participate in the dysregulation of CD (Best et al., 1979; Hollander, 2003; Mayer and Tillisch, 2011). The brain-gut axis refers to a complex bi-directional interactive system comprised of multiple interconnections between the neuroendocrine pathways, the autonomous nervous system, and the gastrointestinal tract. Behavior and brain function may be affected by intestinal inflammation, and vice versa. Therefore, it is of great value to explore the connection between neural changes and inflammatory activity.

The present research used a novel approach of combining MAP-MRI data with regions of interest (ROI)-wise functional connectivity (FC) analysis to uncover brain alterations in CD patients and their relationships with disease activity conditions. We hypothesized that on account of the inseparability of brain structure and function, there were both MAP parameter and FC changes in specific brain regions between these two groups. We also hypothesized that the altered microstructural and functional features would be associated with the levels of inflammatory activity of CD and, hence, may serve as sensitive clinical biomarkers for evaluating disease activity in CD patients.

## Materials and methods

### Participants

This study was approved by the Research Ethics Committee of Renji Hospital Affiliated to Shanghai Jiao Tong University School of Medicine. Written informed consent was obtained from each subject. A total of fifty patients with CD were recruited from April 2020 to January 2022. Inclusion criteria were as follows: (1) right-handed; (2) 18–55 years old; and (3) education level greater than 6 years. Exclusion criteria: (1) intestinal-related abdominal surgery; (2) recent use of glucocorticoids, biologics, or psychotropic medications; (3) organic brain lesions or obvious WM degeneration; and (4) MRI scan-related contraindications such as claustrophobia or metallic implants. Twenty-two patients were finally enrolled in this study after being evaluated and screened by a gastroenterologist with extensive expertise. The medical records of the patients were reviewed throughout the evaluation to acquire endoscopic and hematological data, as well as supplementary information such as disease duration in months.

Twenty-two healthy control subjects (HCs) were recruited via advertisements and were matched for age, gender, education level, and handedness. They had no digestive or pain-related disorders, and their colonoscopy examinations were negative. The inclusion and exclusion criteria for the HC group were identical to those for the patient group.

### Clinical examinations

The serum C-reactive protein (CRP), erythrocyte sedimentation rate (ESR), fecal calprotectin (Calpro), and the Crohn’s Disease Activity Index (CDAI) (Best et al., 1979) were collected to assess the disease condition of each patient, including the degree of inflammatory activity and severity. The CDAI is a globally accepted index for accurately evaluating the disease severity and tentatively predicting the therapeutic outcome of CD patients. CDAI ≤ 150 was considered remission, and CDAI > 150 was considered an active disease. In addition, the disease duration was recorded in months.

### Magnetic resonance imaging data acquisition

All participants were scanned using a 3.0 Tesla MR system (SIEMENS MAGNETOM Prisma) equipped with a 64-channel phase-array head coil. Participants were advised to stay awake throughout the scanning, with their eyes closed and ears muffled, and to avoid thinking about anything in particular. The three-dimensional T1-weighted anatomical images were obtained in the sagittal orientation using the following parameters: TR = 1, 800 ms, TE = 2.28 ms, slice thickness = 1 mm, flip angle = 8°, field of view = 256 × 256 mm^2^, matrix = 256 × 256, and number of slices = 160. The rs-fMRI data were collected using an echo planar imaging sequence with the following parameters (multi-band, acceleration factor = 2): TR = 2,000 ms, TE = 30 ms, slice thickness = 2 mm, flip angle = 90°, field of view = 230 × 230 mm^2^, matrix = 64 × 64, number of slices = 70, and total volume = 220 which was acquired in 7 min and 33 s. The diffusion-weighted images were obtained with the following parameters (multi-band, acceleration factor = 2): TR = 6,800 ms, TE = 75 ms, field of view = 192 × 192 mm^2^, matrix = 128 × 128, number of slices = 92, slice thickness = 1.5 mm, gap = 0. Diffusion-sensitizing gradients were applied along 100 non-collinear directions with a *b*-value of 3, 000 s/mm^2^, and a reference image was acquired at *b* = 0 s/mm^2^.

### Magnetic resonance imaging data preprocessing

Diffusion-weighted images underwent eddy current and motion correction using the DiffusionKit eddy tool (Xie et al., 2016). MAP-MRI parameters were calculated using NeuDiLab, a software based on an open-source tool named DIPY (Diffusion Imaging in Python) (Wang et al., 2021). In a nutshell, the MAP was fit with a radial order of six. A positivity constraint and Laplacian regularization with a weighting of 0.05 were utilized for denoizing during fitting. The ultimate generating parameters were recommended for robust fitting by the developers of the DIPY MAP-MRI toolbox. Finally, maps of MAP-MRI parameters were constructed, encompassing mean square displacement (MSD), non-Gaussianity (NG), non-Gaussianity axial (NGAx), non-Gaussianity vertical (NGRad), Q-space inverse variance (QIV), return to the origin probability (RTOP), return to the axis probability (RTAP), and return to the plane probability (RTPP). They were all normalized to the standard Montreal Neurological Institute (MNI) space in two steps using MRIcron^1^ and then smoothed with a 6 mm full width at half-maximum (FWHM) Gaussian kernel with a reslicing resolution of 2 × 2 × 2 mm^3^.

The rs-fMRI data were preprocessed in MATLAB (MathWorks, Natick, Massachusetts, United States) using the Data Processing and Analysis for Brain Imaging (DPABI) toolkit package (version v4.3).^2^ The main procedures were brain extraction, slice timing correction, rigid-body motion correction, spatial smoothing using a 6 mm FWHM Gaussian kernel, and 150 s high-pass temporal filtering. Moreover, 24 head-motion parameters (6 head motion parameters at baseline, 6 head motion parameters at 1 time point prior, and the 12 associated squared items) were utilized as nuisance variables for regression, along with cerebrospinal fluid and WM signal.

### Group comparison and correlation analysis of mean apparent propagator parameters

A two-sample independent *t*-test was used to compare all of the standardized MAP parameter maps between CD patients and HCs, using the Statistical Parametric Mapping 12 (SPM12)^3^ toolbox in MATLAB. Given the relatively small sample size, a permutation test with a threshold free cluster enhancement (number of permutations = 1,000, *p* < 0.01) was used to extract the brain areas with significant inter-group differences. The automated anatomical labeling (AAL) atlas (Tzourio-Mazoyer et al., 2002) was used to identify the above-mentioned brain regions. The mean values of MAP parameters, including MSD, NG, NGAx, NGRad, QIV, RTAP, RTOP, and RTPP in the CD group, were calculated from the regions exhibiting significant group differences. After adjusting for age, gender, education level, and body mass index (BMI), Pearson’s partial correlation analyses were performed to assess the relationship between clinical characteristics and MAP parameters using SPSS v25 (IBM, Armonk, NY, United States). All *p*-values < 0.05 were considered statistically significant, after false discovery rate (FDR) correction for multiple comparisons.

### Regions of interest-wise functional connectivity analysis

ROIs were defined as brain regions where MAP values were significantly correlated with clinical characteristics. We conducted an ROI-to-ROI FC analysis using the DPABI toolkit. The mean time series for each ROI were extracted using the preprocessed rs-fMRI data. The pairwise FC strength was then computed using Pearson’s correlation coefficients on the time series and Fisher’s *r*-to-*z* transformation to convert the correlation coefficients into z-scores. CD-related changes in FC were evaluated by the group comparison between patients and HCs. All *p*-values < 0.05 were considered statistically significant after FDR correction for multiple comparisons.

## Results

### Demographic and clinical characteristics

Table 1 shows the demographic and clinical characteristics of the two groups. No significant differences were found in age, gender, or level of education between patients and HCs (two-sample *t*-tests and a chi-squared test, all *p*-values > 0.1).

**TABLE 1:** Demographic and clinical characteristics of all participants.

	Patients (*n* = 22)	HCs (*n* = 22)	*P*-value
Age, years	33.60 ± 14.67	37.55 ± 7.43	0.292
Gender (male/female)	12/10	12/10	1.00
Education level, years	12.06 ± 3.23	12.80 ± 1.32	0.487
BMI	19.15 ± 2.93	/	
Disease duration, months	59.78 ± 72.67	/	
CRP, mg/dL	24.52 ± 24.41	/	
ESR, mm/h	38.00 ± 27.70	/	
Calpro, mg/L	323.90 ± 151.18	/	
CDAI	171.12 ± 68.58	/	

Values are represented as the mean ± standard deviation, except for the gender distribution. Two-sample t-tests were performed to assess group differences for age and education, and a chi-squared test for gender. P-value < 0.05 was considered to be statistically significant.

HC, healthy control; BMI, body mass index; CRP, C-reactive protein; ESR, erythrocyte sedimentation rate; Calpro, calprotectin; CDAI, Crohn’s Disease active index.

### Group comparison of the mean apparent propagator parameters

The brain regions with significantly different MAP parameters are shown in Table 2. They are as follows: bilateral parahippocampal gyrus (PHG), bilateral thalamus (THA), bilateral insula (INS), left hippocampus (HIP.L), left putamen (PUT. L), left amygdala (AMYG.L), left temporal pole: superior temporal gyrus (TPOsup.L), left Rolandic operculum (ROL.L), left fusiform gyrus (FFG.L), right middle frontal gyrus (MFG.R), right medial superior frontal gyrus (SFGmed.R), and right anterior cingulate and paracingulate gyri (ACG.R).

**TABLE 2:** Significant differences in the MAP parameters between CD patients and HCs.

MAP parameters	Brain regions	BA	MNI			*t*-value	Voxels
			X	Y	Z		
MAP-MSD	PHG.L	28	−14	−26	−52	4.43	283
	PHG.R	35	26	−14	−30	5.29	119
	HIP.L	−	−30	−10	−16	4.46	83
	PUT.L	34	−28	2	−14	4.73	97
	MFG.R	10	44	58	12	4.37	188
	TPOsup.L	22	−58	10	0	4.51	71
	ROL.L	13	−44	−4	2	5.65	1,140
	SFGmed.R	9	4	56	38	4.01	169
MAP-NG	THA.R	−	8	−18	12	−4.13	109
	ROL.L	13	−38	−2	20	−4.58	178
MAP-NGAx	MFG.R	46	48	54	8	4.12	87
	THA.R	−	8	−18	12	−4.20	126
	ROL.L	13	−38	−2	20	−4.77	218
MAP-NGRad	MFG.R	10	48	54	8	4.05	77
	THA.R	−	16	−18	14	−4.09	75
	INS.L	13	−38	−2	18	−4.09	74
MAP-QIV	PHG.L	35	−14	−18	−48	4.37	395
	PHG.R	35	26	−12	−32	4.50	75
	HIP.L	35	−30	−10	−16	4.32	70
	PUT.L	34	−28	2	−12	5.23	89
	MFG.R	10	44	58	12	4.34	78
	ROL.L	13	−36	−2	16	5.54	884
MAP-RTAP	AMYG.L	34	−26	4	−16	−5.35	91
	FFG.L	36	−22	−40	−12	−5.51	82
	ROL.L	13	−42	−6	2	−5.50	994
MAP-RTOP	AMYG.L	34	−26	4	−16	−5.08	122
	FFG.L	36	−22	−40	−12	−5.12	86
	ROL.L	13	−42	−4	2	−5.44	1,009
	THA.L	−	−14	−24	2	−3.80	65
	THA.R	−	10	−18	6	−4.42	91
MAP-RTPP	MFG.R	10	48	54	8	4.17	90
	PHG.R	35	26	−16	−28	−4.82	98
	PHG.L	28	−22	−16	−26	−4.68	130
	THA.R	27	10	−26	−8	−4.51	449
	HIP.R	13	40	−22	−6	−4.329	73
	INS.L	13	−44	−4	2	−5.78	835
	INS.R	13	32	−20	26	−3.96	82
	ACG.R	24	6	24	20	−4.98	77

The statistical threshold was set at p < 0.01.

BA, Brodmann area; CD, Crohn’s Disease; HC, healthy controls; PHG, parahippocampal gyrus; THA, thalamus; INS, insula; HIP.L, left hippocampus; PUT. L, left putamen; AMYG.L, left amygdala; TPOsup.L, left temporal pole: superior temporal gyrus; ROL.L, left Rolandic operculum; FFG.L, left fusiform gyrus; MFG.R, right middle frontal gyrus; SFGmed.R, right medial superior frontal gyrus; ACG.R, right anterior cingulate and paracingulate gyri.

### Associations between clinical characteristics and mean apparent propagator parameters

**FIGURE 1:**
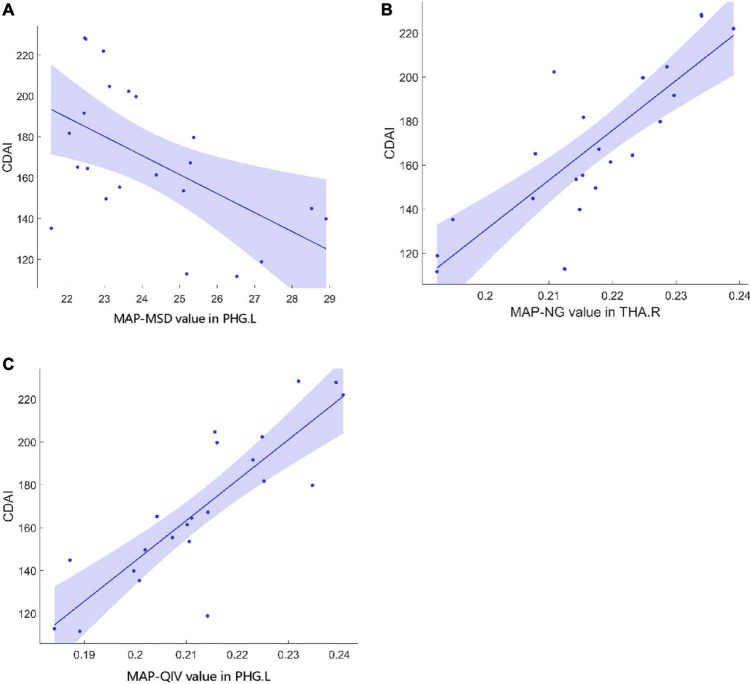
Correlations between MAP parameters and CDAI in CD patients. **(A)** The MSD value in PHG.L (*r* = –0.61, *p* = 0.02) was negatively correlated with CDAI. **(B)** The NG value in THA.R (*r* = 0.53, *p* = 0.019) was positively correlated with CDAI. **(C)** The QIV value in PHG.L (*r* = 0.70, *p* = 0.005) was positively correlated with CDAI. MAP, mean apparent propagator; CDAI, Crohn’s Disease Activity Index; CD, Crohn’s Disease; MSD, mean square displacement; PHG.L, left parahippocampal gyrus; NG, non-Gaussianity; THA.R, right hippocampus; QIV, Q-space inverse variance.

Table 3 shows the brain regions where MAP parameters were significantly correlated with clinical characteristics in patients. The MSD value in the left parahippocampal gyrus (PHG.L) was negatively correlated with CDAI, while the NG value in the right thalamus (THA.R) and the QIV value in the PHG.L were both positively correlated with CDAI (Figure 1). The values of NG, NGAx, RTOP, and RTAP in the ROL.L, and the NGRad value in the left insula (INS.L) were both positively correlated with disease duration. The RTOP value in the left thalamus (THA.L) was positively correlated with CRP. The RTPP value in the right parahippocampal gyrus (PHG.R) was negatively correlated with both ESR and Calpro. (All *p*-values < 0.05, after FDR correction).

**TABLE 3:** Correlations between clinical characteristics and MAP parameters.

		Disease duration	CRP	ESR	Calpro	CDAI
MAP-MSD in PHG.L	*r*	−0.03	−0.33	−0.21	0.01	−0.61
	*P*	0.93	0.26	0.47	0.99	0.02*
MAP-NG in ROL.L	*r*	0.77	0.15	0.24	0.09	0.36
	*p*	0.001**	0.61	0.40	0.77	0.21
MAP-NG in THA.R	*r*	0.08	0.36	0.30	0.05	0.53
	*p*	0.76	0.21	0.29	0.87	0.019*
MAP-NGAx in ROL.L	*r*	0.76	0.09	0.19	0.08	0.36
	*p*	0.001**	0.77	0.52	0.78	0.21
MAP-NGRad in INS.L	*r*	0.74	0.34	0.32	0.17	0.32
	*p*	0.001**	0.23	0.27	0.56	0.27
MAP-QIV in PHG.L	*r*	−0.03	0.30	0.25	0.03	0.70
	*p*	0.91	0.29	0.39	0.92	0.005**
MAP-RTAP in ROL.L	*r*	0.62	−0.14	−0.06	0.21	0.06
	*p*	0.01*	0.63	0.85	0.46	0.84
MAP-RTOP in ROL.L	*r*	0.63	−0.04	0.05	0.18	0.04
	*p*	0.009**	0.88	0.86	0.54	0.90
MAP-RTOP in THA.L	*r*	−0.10	0.60	0.33	0.10	0.02
	*p*	0.71	0.023*	0.25	0.75	0.94
MAP-RTPP in PHG.R	*r*	0.19	0.25	0.56	−0.57	0.23
	*p*	0.47	0.39	0.037*	0.032*	0.44

The statistical threshold was set at p < 0.05.

*p < 0.05, **p < 0.01.

PHG.L, left parahippocampal gyrus; THA.R, right thalamus; THA.L, left thalamus; INS.L, left insula; PHG.R, right parahippocampal gyrus; ROL.L, left Rolandic operculum.

### Functional connectivity analysis

**FIGURE 2:**
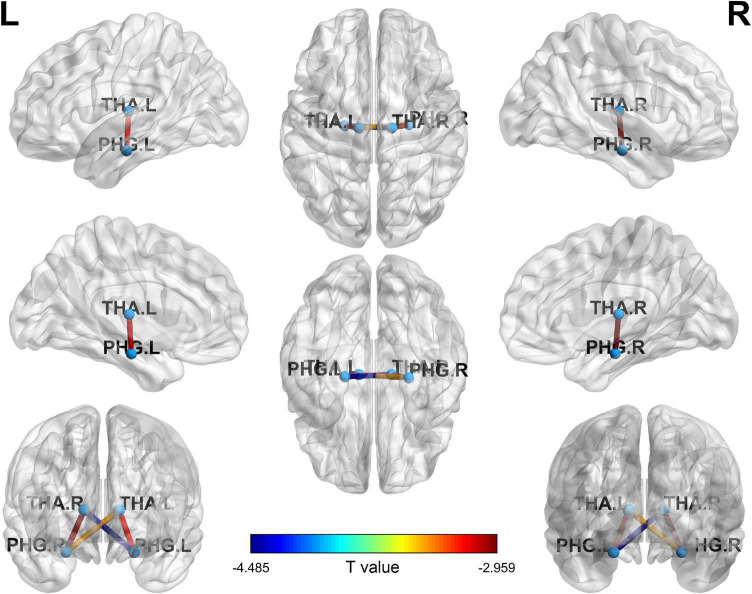
Four ROI to ROI FC significantly decreased in CDs compared with HCs. ROI, region of interest; FC, functional connectivity; CD, Crohn’s Disease; PHG.L, left parahippocampal gyrus; PHG.R, right parahippocampal gyrus; THA.L, left hippocampus; THA.R, right hippocampus.

Four ROI to ROI FC were significantly lower in CD patients compared to HCs. They were THA.L-PHG.L (*t* = −3.117, *p* = 0.034), THA.L-PHG.R (*t* = −3.407, *p* = 0.021), THA.R-PHG.R (*t* = −2.959, *p* = 0.029), and THA.R-PHG.L (*t* = −4.485, *p* = 0.006) FC (Figure 2).

## Discussion

In summary, our study found that the majority of MAP parameters in the parahippocampus and thalamus were significantly different in CD patients from those in the HCs, and that the corresponding FC between the parahippocampus and thalamus were significantly decreased in CD patients.

The MAP-MRI diffusion model, which was recently developed, provides several novel, measurable parameters for capturing previously concealed inherent properties of nervous tissue microstructure. The MSD can be obtained using diffusion propagator models to determine the average amount of diffusion. Zero displacement probabilities, involving the RTOP, RTAP, and RTPP, can be calculated to quantify various features of the three-dimensional diffusion process (Hosseinbor et al., 2013; Özarslan et al., 2013; Ning et al., 2015; Fick et al., 2016). The RTOP is a metric estimated from three-dimensional q-space data, which reflects the probability of water molecules experiencing zero net displacements between the applications of two diffusion sensitizing gradients (Özarslan et al., 2013; Zucchelli et al., 2016). The RTAP and RTPP are two-dimensional and one-dimensional variants of RTOP, respectively. RTAP is decomposed from RTOP perpendicular to the direction of the primary eigenvector and reflects the presence of restrictive barriers in the radial orientation, while RTPP is decomposed parallel to the direction of the primary eigenvector and demonstrates the presence of restrictive obstacles in the axial direction (Özarslan et al., 2013; Zucchelli et al., 2016). Previous studies have shown that MAP indices are clinically applicable in a variety of neuroradiological subspecialties (Avram et al., 2016; Zucchelli et al., 2016; Boscolo Galazzo et al., 2018), and MAP-MRI outperformed the conventional DTI in diagnosing and evaluating Parkinson’s disease (Le et al., 2020). The present study discovered significant differences in MAP parameters between CD patients and HCs in specific brain regions, suggesting the potential value of MAP-MRI in the clinical diagnosis of CD.

Previous studies employing structural and functional MRI have reported some substantial abnormalities in the brain structure and activity of CD patients. Agostini et al. (2013a) showed that CD patients exhibited lower gray matter volume in the frontal lobe and cingulate cortex. Kornelsen et al. (2020), however, found no significant structural difference between CD patients and HCs using voxel-based morphometry analysis, but rather increased FC between the frontoparietal network and the salience network. Prior task-based and rs-fMRI studies have discovered abnormal functional alterations in specific brain areas. In a task-related study, patients with CD had decreased activity in the medial temporal lobe, insula, putamen, and cerebellum (Agostini et al., 2013b). In addition, another study using the stress-evoking task found that CD patients had increased activation in the midcingulate cortex (Agostini et al., 2017; Khairnar et al., 2017). A study using intra-network FC analysis with rs-fMRI data showed significantly increased connectivity within the executive control network and default mode network (Hou et al., 2019). According to Fan et al. (2020), the intrinsic FC between the amygdala and insula, parahippocampus, and anterior middle cingulate cortex significantly decreased and was correlated with the disease duration.

In our study, patients with CD displayed microstructural abnormalities in the PHG and thalamus, as well as decreased FC between them. The PHG and thalamus are two knots of the limbic system that have long been well-established as critical components in affect and emotion processing. Some evidence indicates that CD patients often suffer from mood disorders, overreaction to stress, deficiency in concentration, and gut flora imbalance (Chen et al., 2020), precipitating mental symptoms such as anxiety and depression. Holland and Goadsby proposed that the limbic system structures had an effect on the pain matrix and were particularly involved in nociception inhibition (Yeung, 2021). The PHG is involved in the familiarity and retrieval process of memory. The thalamus acts as a relay station, receiving sensory input and relaying it to the cortex. The decreased FC between these two regions might be a distinctive feature of CD in pain or pain context recollection. Along with the decreased FC between the PHG and thalamus, Fan et al. (2019) demonstrated that CD patients with significantly higher self-reported anxiety or depression had increased FC between the amygdala, thalamus, and orbitofrontal cortex. Both previous research and our findings indicate that abnormal FC within the limbic system could contribute to emotional symptoms in CD patients (Bao et al., 2018).

CD, as a chronic IBD, primarily affects the gastrointestinal tract. Some recent studies have proposed that the microbiota-gut-brain axis may be a critical contributor in understanding the concomitant mental health issues in IBD and related disorders (Lv et al., 2018; Gracie et al., 2019; Thomann et al., 2019; Zhang et al., 2022). Animal-model research showed that chronic gastrointestinal inflammation might induce anxiety-like behavior and alter biochemistry of the central nervous system in mice (Mogilevski et al., 2019). Furthermore, gastrointestinal inflammation is associated with hippocampal changes in inflammatory gene expression and glutamatergic transmission, resulting in increased brain excitability and cognitive impairments (Bonaz and Bernstein, 2013). Serotonin is suspected to play a vital role in gut-to-brain transmission. An immunohistochemical study noted that serotonergic fibers connecting the thalamus to the cortex may exert a considerable influence on affective function (Lv et al., 2017). All of these factors might account for our observations of altered microstructure in the hippocampus and thalamus of CD patients.

There were several limitations to our study. First, the sample size is too small, and it would be preferable to incorporate more clinical information, including manifestations and treatments, and further studies can be conducted to explore the relationship between the clinical manifestations and brain function changes, such as whether the results are consistent between active and remission stages of the disease, or whether they are reversible between courses. Second, now that the thalamus and PHG are pivotal parts of the classical hippocampal circuit within the limbic system, further studies using multimodal MRI can be conducted to investigate the structural and functional network (e.g., small world network) alterations related to emotion processing. Third, since a lot of new brain function templates have admittedly emerged in recent years, additional research is necessary to use other brain templates or atlases including but not limited to the High Angular Resolution Diffusion Imaging (HARDI) template (Varentsova et al., 2014) based on the International Consortium for Brain Mapping (ICBM)-152 space, to concentrate on more complex neuronal microarchitecture, which may yield different and more intriguing findings.

In summary, the altered microstructure and FC of the brain reveal abnormal brain activity in CD patients, which may be related to the pathophysiological mechanism of the disease through the brain-gut axis. The impairment of parahippocampus gyrus and thalamus may serve as a neurological biomarker for disease activity.

## Data availability statement

The raw data supporting the conclusions of this article will be made available by the authors, without undue reservation.

## Ethics statement

The studies involving human participants were reviewed and approved by the Research Ethics Committee of Renji Hospital Affiliated to Shanghai Jiao Tong University School of Medicine. The patients/participants provided their written informed consent to participate in this study. Written informed consent was obtained from the individual(s) for the publication of any potentially identifiable images or data included in this article.

## Author contributions

YQ and QL contributed to the conceptualization, formal analysis, investigation, methodology, and writing – original draft. DW contributed to the review and editing of the manuscript. YiZ performed the formal analysis. JC performed data curation and investigation. ZC and YaZ contributed to the supervision of the manuscript. All authors contributed to the article and approved the submitted version.

## Footnotes

1
http://www.sph.sc.edu/comd/rorden/mricron/

2
http://rfmri.org/dpabi

3
http://www.fil.ion.ucl.ac.uk/spm/software/spm12/
